# Effects of sample size on robustness and prediction accuracy of a prognostic gene signature

**DOI:** 10.1186/1471-2105-10-147

**Published:** 2009-05-16

**Authors:** Seon-Young Kim

**Affiliations:** 1 Medical Genomics Research Center, KRIBB, 111 Gwahangno, Yuseong-gu, Daejeon 305-806, Republic of Korea

## Abstract

**Background:**

Few overlap between independently developed gene signatures and poor inter-study applicability of gene signatures are two of major concerns raised in the development of microarray-based prognostic gene signatures. One recent study suggested that thousands of samples are needed to generate a robust prognostic gene signature.

**Results:**

A data set of 1,372 samples was generated by combining eight breast cancer gene expression data sets produced using the same microarray platform and, using the data set, effects of varying samples sizes on a few performances of a prognostic gene signature were investigated. The overlap between independently developed gene signatures was increased linearly with more samples, attaining an average overlap of 16.56% with 600 samples. The concordance between predicted outcomes by different gene signatures also was increased with more samples up to 94.61% with 300 samples. The accuracy of outcome prediction also increased with more samples. Finally, analysis using only Estrogen Receptor-positive (ER+) patients attained higher prediction accuracy than using both patients, suggesting that sub-type specific analysis can lead to the development of better prognostic gene signatures

**Conclusion:**

Increasing sample sizes generated a gene signature with better stability, better concordance in outcome prediction, and better prediction accuracy. However, the degree of performance improvement by the increased sample size was different between the degree of overlap and the degree of concordance in outcome prediction, suggesting that the sample size required for a study should be determined according to the specific aims of the study.

## Background

Recent advances in various high-throughput technologies including genome sequencing, transcriptomics, genome-wide SNP analysis, proteomics, glycomics, and metabolomics have opened up new opportunities for developing prognostic and predictive markers for better treatment of diverse diseases. Indeed, many researchers have reported promising results for improved patient treatment by providing more accurate prognostic and predictive information for decision making [1-3]. Among various high-throughput technologies, microarray gene expression profiling has been widely used for prognostic and predictive marker development for its rich information. The use of gene expression profiling has particularly been widespread in cancer research and now a few products are already in market for clinical use and there are also a few large scale clinical trials to determine the effectiveness of gene expression profiling as a prognostic marker for cancer patients [2,4-7].

While many researchers have shown promising results on the possibility of gene expression profiling as a prognostic marker, there are also concerns on the hasty use of the technology in the clinic because many issues remain unresolved and some promising research results were presented in an over-optimistic and flawed manner [8-10]. Unresolved issues include the instability of identified prognostic gene signatures, few overlap between independently developed prognostic gene signatures, and poor inter-study applicability of gene signatures [9,11,12]. Here, the instability represents a phenomenon in which prognostic signatures strongly depend on the selection of patients in random sampling processes [9]. Genes repeatedly selected during random sampling are defined as robust here.

Among the above-listed problems, the instability and few overlap of already reported prognostic signatures have received great attention. At first, the few overlap between independently developed gene signatures was attributed to the differences in patients, microarray platforms, or applied statistical analyses. However, Ein-Dor et al. showed that many equally efficient but non-overlapping prognostic gene signatures can be identified from a single data set because gene expression data contains numerous informative genes [11]. Michiels et al. showed that only a few genes are consistently selected from a given data set when they applied random sampling approach in their analysis [9]. To understand the nature of the instability of prognostic gene signatures, Ein-Dor et al. developed a new mathematical model and concluded that at least thousands of samples are needed to develop a stable gene signature [12].

Currently, most gene expression profiling studies have been performed with some tens to hundreds of samples. Meta-analysis, by combining the results of several studies, makes it possible to overcome the limits of many small sample-sized studies. In this work, we pooled eight large-scale gene expression studies to attain a data set with more than 1,300 samples. Specifically, we only used data sets produced using a single microarray platform, Affymetrix U133A, in pooling different data sets to exclude data loss and confounding factors arising from the combination of different microarray platforms. Using more than 1,300 samples, we performed several analyses to understand the various aspects of prognostic gene signatures.

## Results

### Construction of a single data set by pooling eight data sets

**Figure 1:**
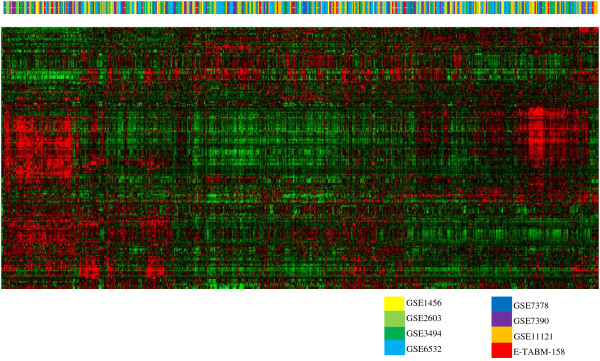
**Pattern of clustering of 1,418 samples from eight data sets**. Each data set was mean-centered and pooled into a single data set of 1,418 samples. Each color above the heatmap represents each data set.

To understand the effects of a sample size on the classifier performances, we first constructed a single data set by pooling eight publicly available breast cancer data sets (Table 1; [13-21]). Several methods including simple mean-centering [22], distance weighted discrimination [23], and empirical Bayes methods [24] are available for adjusting batch effects when combining multiple gene expression data sets. One recent study showed that simple mean-centering can effectively remove many data set specific biases allowing effective integration of multiple data sets [22]. Thus, we applied a simple mean-centering method to the eight data sets and performed clustering analysis to see if any data set specific batch effects are observed in the pooled data set. No distinct batch effects were found in the pooled data set (Figure 1), suggesting that simple mean-centering was able to remove most, if not all, batch-specific biases. Principal Component Analysis (PCA) of the pooled samples again confirmed that batch effects were rarely found in the pooled data set (Additional data file 1). The pool data set was used in the subsequent analyses.

**Table 1:** Data sets analyzed in this study

Data set	Total	ER+	ER-	Survival	Reference
GSE1456	159	99	40	RFS	[13]
GSE2603	82	57	42	DMFS	[14]
GSE3494	236	213	34	DMFS	[15]
GSE6532	306	262	45	DMFS	[16,17]
GSE7378	54	54	0	DMFS	[18]
GSE7390	198	134	64	DMFS	[19]
GSE11121	129	200	0	RFS	[20]
E-TABM-158	344	84	46	DMFS	[21]

Total	1418	1103	271		

### Increased sample size increases overlap between gene sets

**Figure 2:**
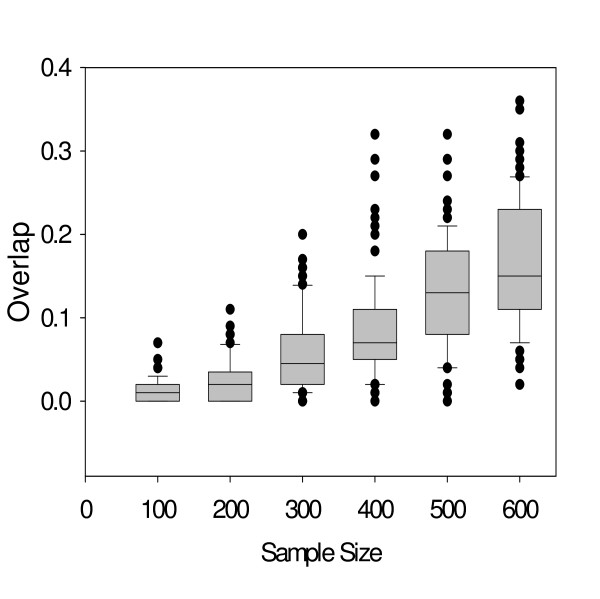
**An overlap between two prognostic gene-sets increases with an increasing sample size**. From a data set of 1,372 samples, n samples were randomly selected and a prognostic gene set was prepared by selecting top 100 genes with the lowest p-value from Cox proportional hazard survival analysis. The sample size n was varied from 100 to 600 by an increment of 100, and the random sampling was performed 200 times for each sample size n. An overlap between two gene-sets was computed for each pair of 200 prognostic gene sets and the distribution of the overlaps was shown as boxplots.

We first calculated the degree of overlap between different prognostic gene signatures as the sample size was varied. An overlap between different prognostic gene signatures increased according to the increased sample size (Figure 2). For example, the average overlap between data sets with 100 samples was 1.33%, but it was increased to 16.56% with 600 samples. This number is in good agreement with Ein-Dor et al. [12]'s prediction which suggested that approximately five to eight hundred samples are needed to attain an overlap of 20% in breast cancer data sets.

### Increased sample size decreases the error rate of class prediction

We then tested the effects of a sample size on the error rate of class prediction which is the most important measure of prognostic classifier performance in clinical decision making [25,26]. For class prediction, each patient was divided into good (relapse or distant metastasis free survival over five years) or poor (relapse or distant metastasis within five years) prognosis groups. Relapse or metastasis free patients followed up less than five years were excluded from the analysis.

We applied random sampling approach in our evaluation of error rate of class prediction by randomly selecting n training samples (from 100 to 500 by an increment of 100) from the pooled data set, constructing a prognostic classifier from the training samples, and evaluating its performance on the 100 randomly selected testing samples [9]. We used three well-established machine learning algorithms – Diagonal Linear Discriminant Analysis (DLDA), Support Vector Machine (SVM), and Random Forest (RF) – in our analysis [27]. While SVM and RF algorithms need fine tuning of several parameters to attain the lowest error rate of prediction, we just applied default parameters given in the R packages (e1071 for SVM and RandomForest for RF) because we had to perform numerous class predictions on several hundred data sets prepared by re-sampling at each sample size.

**Figure 3:**
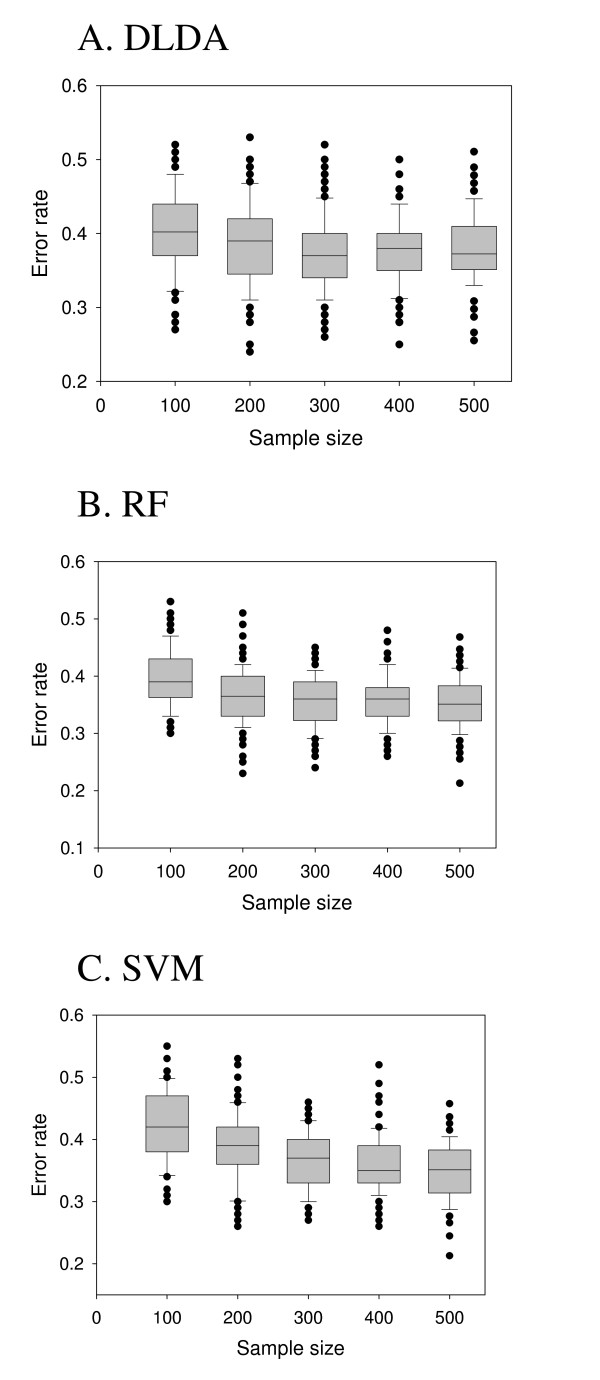
**The error rate of prediction decreases with an increasing training sample size**. A. DLDA, B. RF, C. SVM. First, each sample was labeled as good (disease-free or overall survival over five years) or poor (recurrence or death within five years). Then, m training samples and 100 testing samples were randomly selected from the data set of pooled samples, a prognostic gene set was constructed from the m training samples, and its error rate of prediction was calculated by applying the prognostic gene set to the 100 testing samples. The training sample size m was varied from 100 to 500 by an increment of 100, and the entire process was repeated 100 times. Three machine learning algorithms – DLDA, RF, and SVM – were used. Data represents a boxplot of error rates calculated by 100 random sampling processes.

The error rate of class prediction was decreased as the number of training samples used for constructing prognostic gene signatures increased (Figure 3) with all the three algorithms producing similar results. The best average error rate was 34.66% obtained from the training sample size of 500 with support vector machine algorithm.

### Concordance between predicted outcomes increases with an increasing training sample size

**Figure 4:**
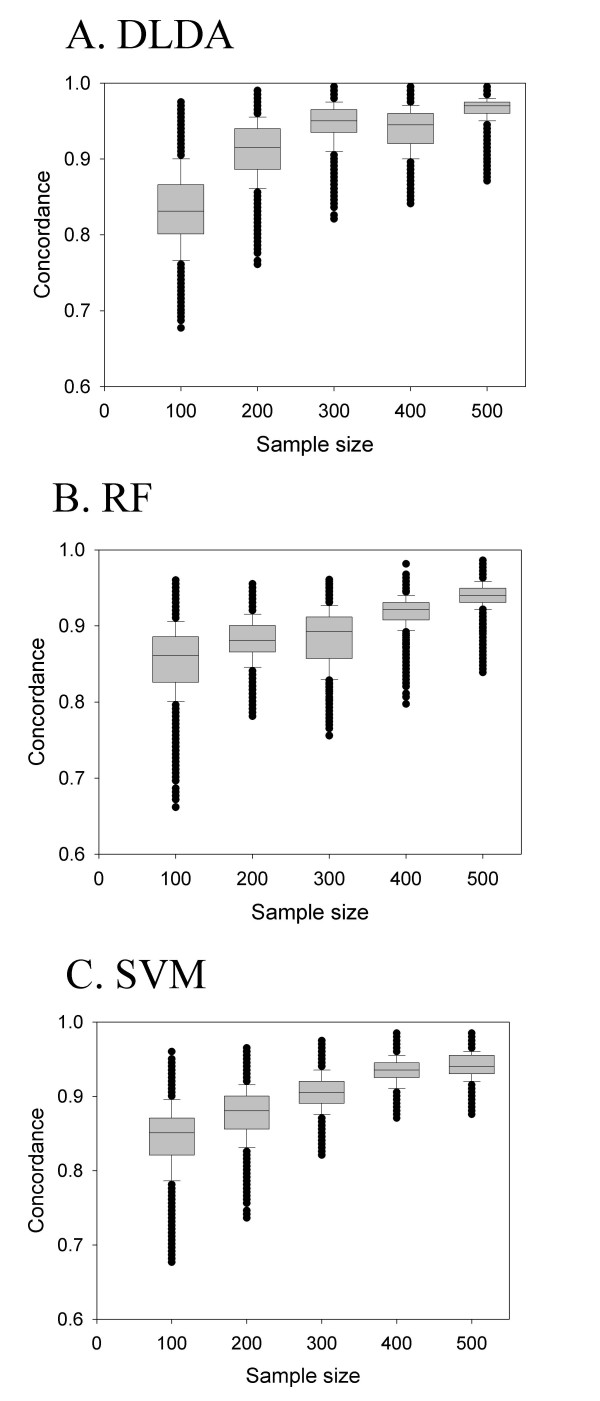
**Concordance between predicted outcomes increases with an increasing training sample size**. For each sample size from 100 to 500 by increments of 100, one hundred samples were first selected as testing samples and 100 independently selected training samples were used to predict the outcomes of the already selected testing samples. Concordance of outcome prediction between each pair of 100 predictions (a total of 4950 pairs) was calculated. Three different algorithms (A. DLDA, B, RF, and C. SVM) were tested.

Recently, Fan et al. emphasized that concordance in the predicted outcomes between different prognostic gene signatures is the more relevant measure than the mere overlap between them in evaluating the similarity between different gene signatures [28]. We thus investigated the effects of different training sample sizes on the concordance in the predicted outcomes. For each sample size, 100 samples were first left out as testing samples, and n samples were randomly selected from the remaining samples to produce a prognostic gene signature. The random sampling process was repeated 100 times to produce 100 independent prognostic gene signatures. For each of the 100 independently prepared prognostic gene signature, outcomes were predicted on the 100 initially left-out testing samples and concordance in the predicted outcomes among the 100 gene signatures were measured. As expected, the concordance in the predicted outcomes increased as the training sample size was increased (Figure 4). For example, the mean concordance was 83.3% at a training sample size of 100, but it was increased to 91.16% with 200 training samples, and further increased to 96.52% with 500 training samples. Similar patterns were found with SVM and RF algorithms.

### Sub-type specific gene signature decreases the prediction error rate

Recent studies have shown that breast cancer is a heterogeneous disease consisting of three to six different molecular subtypes [29,30]. The estrogen receptor (ER) status is one of the important molecular phenotypes in classifying breast cancers into different subtypes [31]. Until now, to increase the total sample size, we didn't divide samples into different ER groups (ER-positive and ER-negative). To see if sub-type specific analysis could improve the performance of prognostic gene signatures, we first divided samples into ER-positive and ER-negative groups and performed analysis using only the ER-positive samples. ER-negative sample specific analysis was not performed due to the small number of ER-negative samples (Table 1).

**Figure 5:**
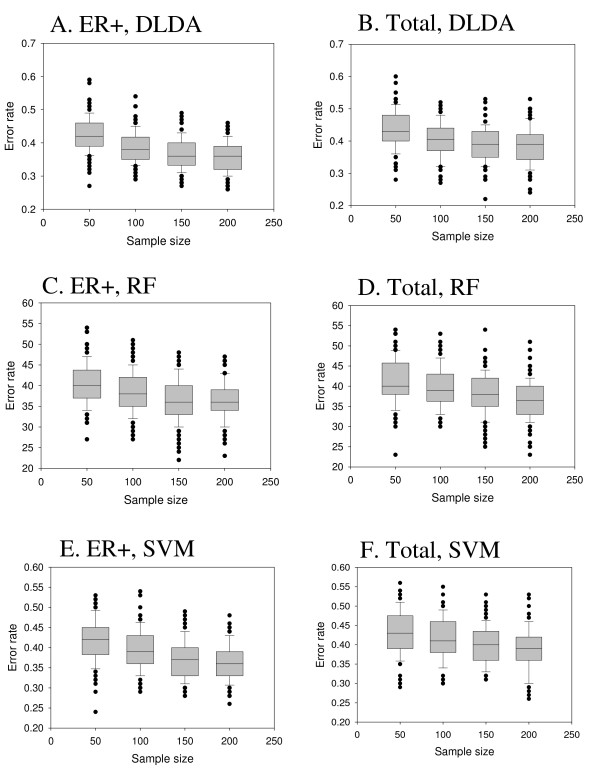
**Sub-type specific gene signature decreases the prediction error rate**. Estimation of prediction error rate by random sampling of training-testing samples was restricted to Estrogen-Receptor positive (ER+) samples, and its error rate (ER+ only) was compared with that of total (both ER+ and ER-) samples. A. ER+ samples by DLDA, B. Total samples by DLDA, C. ER+ samples by RF, D. Total samples by RF, E. ER+ samples by SVM, F. Total samples by RF.

With ER-positive samples, the number of training sample was varied from 50 to 200 with an increment of 50. As expected, analysis using only ER-positive samples always produced lower error rates of prediction that the analysis using both ER-positive and ER-negative samples did (Figure 5). For example, using DLDA algorithm, an average prediction error rate of 35.92% was achieved by 200 samples in ER-positive specific analysis in comparison to an average error rate of 38.71% in an analysis using both ER-positive and ER-negative samples (P < 0.000224 by unpaired *t*-test).

## Discussion

Using more than 1,300 samples prepared by pooling eight independent data sets, we explored the effects of a sample size on three metrics: the degree of overlap between independently developed gene signatures, the accuracy of outcome prediction, and the degree of concordance in outcome prediction between independently developed gene signatures. We also tested if the accuracy of outcome prediction could be further improved by sub-type specific analysis. We found that all the three metrics were improved by the increased sample size, but in different degrees.

The degree of an overlap between independently developed gene signatures increased in proportion to the number of training samples. With a sample size of 600, a mean of 16.56% overlap was observed (Figure 2), which is in good agreement with the results of Ein-Dor et al. who showed that 500–800 samples are needed for 20% overlap and approximately 2000–3000 samples are needed for 50% overlap [12]. Thus, Ein-Dor et al.'s prediction is well supported by a real gene expression data set in our analysis. The same conclusion was obtained by Vliet et al. [32] who showed that small sample size problem is the most relevant explanation for the poor overlap between small-sized data sets. The increased sample size will typically increase overlap between independently developed gene signatures by reducing variability between classifiers from random sampling.

However, when we turned our focus on the concordance in outcome prediction between different gene signatures, we found that 200–300 samples were enough to achieve reasonably good performance. For examples, with DLDA algorithm, 91.16% and 94.61% concordant outcome predictions were achieved with 200 and 300 samples (Figure 3A), and similar results were obtained with RF and SVM algorithms (Figure 3). The discrepancy between the degree of overlap and the degree of concordance in outcome prediction improved by increased sample size suggests that the two measures of the performance of prognostic gene signatures may be unrelated to each other [16,33]. Dobbin et al. recently emphasized that the identification of a gene signature with optimal prediction accuracy should be distinguished from the identification of a robust gene signature and that thousands of samples may not be needed to produce a good classifier [20]. In another study, Fan et al. showed that the lack of overlap between different gene signatures may not be as serious a problem as originally thought if different gene signatures are concordant in their outcome prediction and represent similar biological processes and pathways [16,20]. That Ein-Dor et al. could develop as many as eight independent, but equally prognostic gene signatures from a single data set gives another support for the view of putting little importance on the overlap between different signatures [11,21]. Because high-throughput gene expression data contain enormous amounts of information and many genes are co-regulated, it is comprehensible that many equally efficient gene signatures can be developed from a single data set [16,20].

Many morphologically similar tumors are heterogeneous at the molecular level. For example, recent gene expression profiling studies have established that breast cancer can be divided into three to six molecular subtypes by the pattern of gene expression [29,30,34]. The ER status is the most important molecular character to classify breast cancers into sub-types and many studies have shown that ER-positive breast cancer is fundamentally different from ER-negative one and should be treated differently [29,31]. For this reason, we tested if developing prognostic gene signatures in a sub-type specific manner could further improve the prediction accuracy of a gene signature. Results showed that about 3–5% improvement in prediction accuracy is obtained by developing ER+ specific gene signatures. Many recent works report the development of ER+ or ER- specific gene signatures with much improved performance [35,36].

We acknowledge that our work has several points for improvement. First, survival information, which was arbitrarily dichotomized into binary outcomes for a class prediction problem, may be used as a continuous variable for its full use. Second, while we used only the ER-status variable in our sub-type specific analysis of breast cancer data sets, other clinical attributes such as node status, grade, age, or treatment status should be considered as confounding factors in the analysis.

## Conclusion

Increasing sample sizes generated a gene signature with better stability, better concordance in outcome prediction, and better prediction accuracy. However, the degree of performance improvement by the increased sample size was different between the degree of overlap and the degree of concordance in outcome prediction. Thus, while thousands of samples might be needed to achieve 50% or more overlap, 200–300 samples were enough to achieve between 90 and 95% concordance in outcome prediction. Finally, sub-type specific analysis produced better results suggesting that developing prognostic gene signatures for specific patient sub-groups (i.e. ER-positive and negative breast cancer patients, respectively) may be a better strategy for heterogeneous diseases such as breast cancer.

## Methods

### Datasets and preprocessing of microarray data

Eight breast cancer gene expression data sets with clinical information on patient survival and CEL files were obtained from the Gene Expression Omnibus (GEO) [37] or ArrayExpress [38]. See Table 1 for a complete list of data sets and their sources. Only data sets generated using the Affymetrix U133A platform were included. Each data set was uniformly processed by RMA algorithm using the downloaded CEL files, mean-centered, and then pooled together into a single data set of 1,418 samples. Clustering of the 1,418 samples was performed to see if there were any batch effects among the eight combined data sets. Then, 46 samples in which survival information is missing were excluded resulting in a total of 1,372 samples in subsequent analyses.

### Overlap between prognostic gene sets obtained from random sampling approach

From the data set of 1,372 samples, n samples were randomly selected without replacement and a prognostic gene set was built from the n samples by selecting top 100 genes with the lowest p-value from Cox proportional hazard survival analysis. The sample size n was varied from 100 to 600 by an increment of 100 and the random sampling was repeated 200 times for each sample size n. For each sample size, an overlap between each pair of 200 prognostic gene sets was calculated [9].

### Prediction accuracy

First, each patient was divided into good (relapse or distant metastasis free survival over five years) or poor (relapse or distant metastasis within five years) prognosis groups. Relapse or metastasis free patients followed up less than five years were excluded from the analysis.

Three widely used machine learning algorithms, Diagonal Linear Discriminant Analysis (DLDA), Support Vector Machine (SVM), and Random Forest (RF), were used in the analysis [15]. For each prognosis group, n training and 100 testing samples were randomly selected, a prognostic predictor was constructed from the n training samples, and its prediction accuracy was assessed by applying the predictor on the 100 testing samples. For all the three algorithms, genes differentially expressed between good and poor prognosis groups (p < 0.001 by *t*-test) were first selected and then used in the subsequent analyses. The training sample size n was varied from 100 to 500 by an increment of 100 and the random sampling was performed 100 times for each sample size. An average of prediction error rates from the 100 random sampling was reported for each sample size. An equal number of samples were selected from the good and poor groups during random sampling of a training-testing pair to avoid a bias in error rate estimation that occurs when the sizes of two classes are severely unbalanced. For the analysis of ER+ specific data set, the training sample size n was varied from 50 to 200 by an increment of 50. The R statistical programming language (version 2.6.2) [39] and Python programming language (version 2.5.2) [40] were used for statistical analyses and data manipulation, respectively. The e1071 package (for SVM, version 1.5–18) and the randomForest package (for RF, version 4.5–25) were obtained from the comprehensive R archive network (CRAN) website and the DLDA algorithms were implemented using the Python programming language. To briefly describe DLDA, it is relatively simple but efficient linear rule based on the maximum likelihood discriminant rule [41]. In DLDA, a sample is assigned to a class k in which



is minimized, where p is the number of genes, *x*_*j *_is the value on gene j of the test sample,  is the sample mean of class k and gene j, and σ_j_^2 ^is the variance of the gene [27]. For a brief description of SVM and RF, please see Diaz-Uriarte et al., too [27]. For SVM, radial-basis kernel with a gamma value of one over the number of columns was used. For RF, the number of trees to grow was set to 200, cases were sampled with replacement, and mtry (number of variables randomly selected as candidates at each split) was set to square root of training sample size.

### Analysis of the concordance in the outcome prediction

The effect of a sample size on the concordance in the outcome prediction between different prognostic gene sets was analyzed as follows. Five different sample sizes from 100 to 500 by an increment of 100 were used in the analysis. For each sample size, we first left out 100 testing samples from the total samples for outcome prediction. Then, n training samples were randomly selected from the remaining samples and used to produce prognostic gene signatures. For each sample size, one hundred random samplings were performed to produce 100 independent prognostic gene signatures and each signature was used to predict the outcomes of the left-out test samples at the first step. The concordances in the predicted outcomes for each pair among the 100 prognostic gene signatures were calculated [16].

## List of abbreviations used

GEO: Gene Expression Omnibus; GSE: Gene expression Series; DLDA: Diagonal Linear Discriminant Analysis; SVM: Support Vector Machine; RF: Random Forest;

## Authors' contributions

SYK designed the study, collected datasets, performed bioinformatics analyses, and wrote the manuscript.

## Supplementary Material

Additional File 1 **Two-dimensional PCA (Principal Component Analysis) plot of the pooled samples**. Each color represents the eight different data sets and each point represents different samples. Cluster program was used for PCA analysis and the xyplot function of the lattice graphics package of R was used for plotting. Click here for file
