# Supplementary figures and images for: Effects of sample size on robustness and prediction accuracy of a prognostic gene signature
Source: BMC Bioinformatics. 2009 May 16;10:147. doi: 10.1186/1471-2105-10-147 (PMC2689196; doi:10.1186/1471-2105-10-147)

## Slide 1
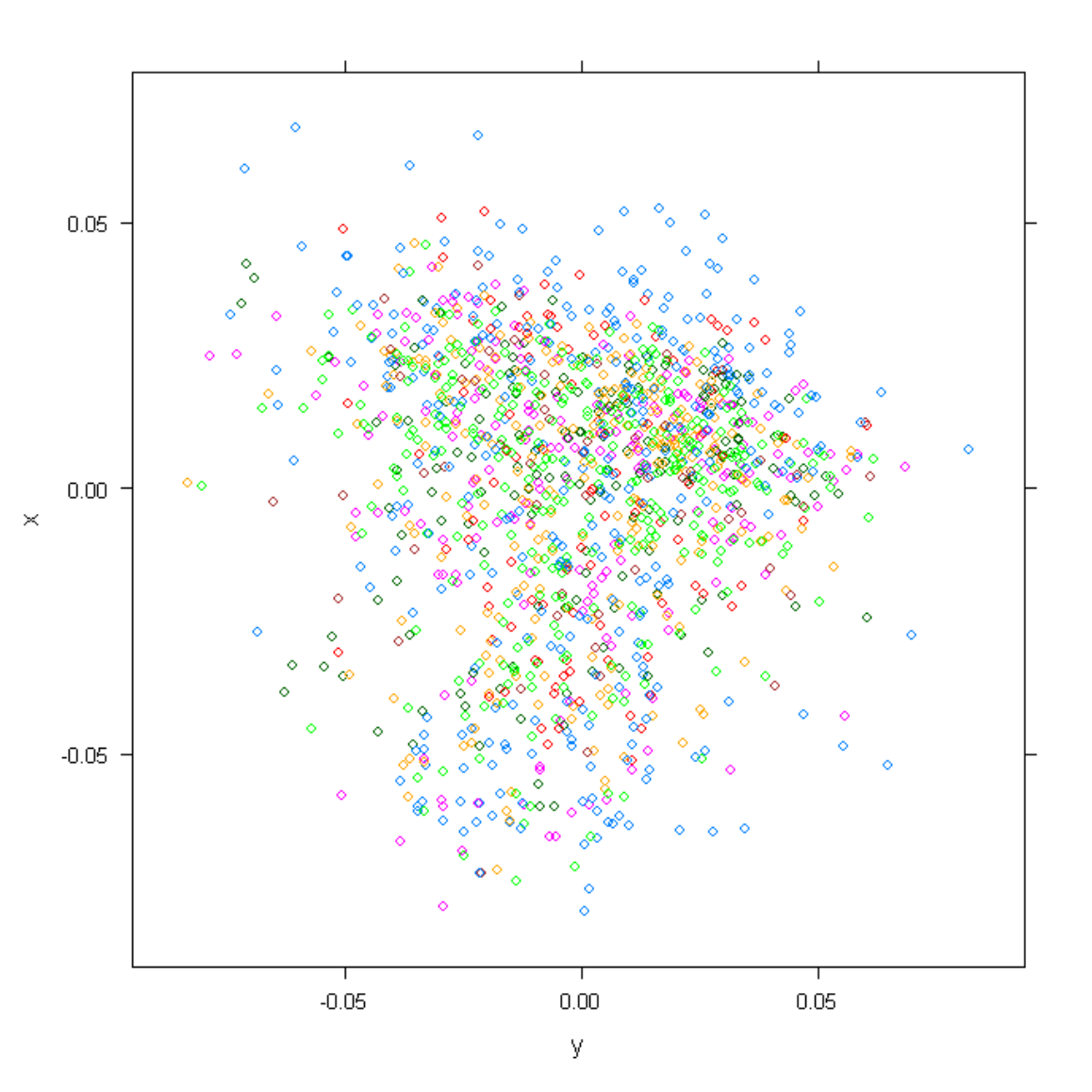

Supplement: Additional File 1 — Two-dimensional PCA (Principal Component Analysis) plot of the pooled samples. Each color represents the eight different data sets and each point represents different samples. Cluster program was used for PCA analysis and the xyplot function of the lattice graphics package of R was used for plotting. [file 1471-2105-10-147-S1.ppt]
